# Information needs of children with leukemia and their parents’ perspectives of their information needs: a qualitative study

**DOI:** 10.1186/s12887-022-03478-w

**Published:** 2022-07-13

**Authors:** Noyuri Yamaji, Yasuko Nagamatsu, Kyoko Kobayashi, Daisuke Hasegawa, Yuki Yuza, Erika Ota

**Affiliations:** 1 Global Health Nursing, Graduate School of Nursing, St. Luke’s International University, 10-1 Akashi-cho, Chuo-ku, Tokyo, 104-0044 Japan; 2 Child Health Nursing, Graduate School of Nursing, St. Luke’s International University, 10-1 Akashi-cho, Chuo-ku, Tokyo, Japan; 3 Department of Pediatrics, St. Luke’s International Hospital, 9-1 Akashi-cho, Chuo-ku, Tokyo, 104-0044 Japan; 4 Department of Hematology and Oncology, Tokyo Metropolitan Children’s Medical Center, 2-8-29 Musashidai, Fuchu-shi, Tokyo, 183-8561 Japan

**Keywords:** Child, Parent, Leukemia, Information needs, Qualitative research

## Abstract

**Background:**

Despite the potential benefits of effective communication, telling a child that they have a life-threatening condition is one of the most daunting challenges. This study aimed to explore the information needs of children with leukemia from the perspectives of children and their parents at the time of diagnosis.

**Methods:**

We conducted an exploratory qualitative study using semi-structured individual interviews with children diagnosed with leukemia between seven and 13 years old (*n* = 7) and their parents (*n* = 9). Children and parents’ interview data were analyzed using thematic analysis.

**Results:**

We identified three themes for the information needs of children with leukemia, 1) beginning to cope, 2) avoiding disclosure – protecting child, and 3) informational support. The children and their parents needed to receive understandable information at the best time to cope with cancer. However, the children and parents expressed different views about children’s information needs. The children needed clear information about the disease, treatment, hospitalization, and the benefits of hospitalization from the time of diagnosis. In contrast, the parents felt they should not tell their children about the disease if they were in shock by their child’s cancer diagnosis. Moreover, the parents believed that information that would be incomprehensible to the child and distress should be avoided to protect their children.

**Conclusions:**

While the information needs of children with leukemia are varied, children and their parents need the information to cope with cancer. However, if the parents believe that the information would be distressful, they might manage communication with their children. Healthcare professionals should explore the motivations behind parents’ attitudes against communication with children and confront conflict. Healthcare professionals also should communicate with the children and their parents to understand their information needs and respect children’s views.

## Background

Approximately 285,000 children between the ages of 0 and 14 are diagnosed with cancer annually worldwide [1], including 2000 in Japan. Cancer is one of the top causes of children’s death in Japan [2], and leukemia is the most common type of childhood cancer [1]. Children diagnosed with cancer face a great health threat, and their lives are changed immediately after diagnosis. The experience of childhood cancer might be a stressful and traumatic experience [3]. Some previous guides and reports suggested that effective communication supports and facilitates children’s and parents’ adjustment to diagnosis [4]. Information is necessary for children with cancer to understand their situation correctly and adopt actions supportive of their goals [5, 6]. Moreover, having sufficient and clear information decreases anxiety and depression [7], and helps to maintain good relationships among children, their families, and healthcare professionals (HCPs) [8].

Despite the potential benefits of effective communication, telling a child that they have a life-threatening condition is one of the most daunting challenges for the HCPs and families [7]. Many children are not told about their diagnosis [7], and 40% of physicians did not talk about a cancer diagnosis to children aged six to 9 years old in Japan [9]. Although children’s and parents’ preferences are sometimes different [10, 11], most studies on communication in childhood cancer have focused on parents or physicians rather than children [12]. While there have been studies examining the information needs of parents [13–15], few studies focused on the preferences of children’s information needs. Also, child-focused guidelines about communication with children with cancer are limited globally [7, 16]. It is necessary to understand children’s information needs from both perspectives and offer relevant information [17].

## Methods

### Aim

This study aimed to explore children’s information needs when diagnosed with leukemia from the perspective of children and their parents. The findings could inform HCPs and families to help children with leukemia receive appropriate information and be better equipped to cope with leukemia.

### Study design

We used an exploratory qualitative design to surface the information needs of participants. This qualitative study used data from semi-structured interviews with children with leukemia and their parents.

### Setting and participants

The site of the study was a pediatric oncology outpatient unit in Japan. The age limitation criterion was based on Piaget and Lev Vygotsky’s developmental stages [18]. We recruited children with leukemia along with their parents. Children’s inclusion criteria were: 1) diagnosed with cancer, 2) aged between seven and 15 years old at the time of recruitment, 3) diagnosed at the age of three to 12 years old, 4) at least 1 month and within 5 years after the diagnosis, and 5) could understand and speak Japanese. Inclusion criterion for parents was aged over 20 years old. We excluded the participants who had mental disorders.

### Recruitment

We recruited the participants between September 2019 and March 2020. Braun and Clarke, 2013 recommended six to ten participants for interviews [19]; recruitment ended when no new information was discovered that related to the emergent theme. The researcher attended the meetings held the day before the patients’ outpatient visit. With the assistance of the oncologists and nurses, we selected eligible patients who met our criteria. All eligible patients and parents were told the purpose of the study and were invited to participate. The researcher explained the research using the consent form for parents who had confirmed their intention to participate in the study. We also explained all items verbally to children who obtained the consent of their parents, and obtained written informed assent from them.

#### Data collection

We developed an interview guide based on previous studies, to identify children’s communication preferences with cancer and their parents [20, 21] (Table 1). The first author, with 12 years of experience caring for children with cancer, individually interviewed and adapted the questions and order of questions into a conversation with the participants. For example, the child was asked, “Tell me about how and what do you want to know about your disease?” Parents were asked, “Tell me how and what you should tell your child about the disease?” The interviewer was not involved in the care of the participants. The interviews lasted 25 to 70 minutes (mean time: 47 minutes) in an outpatient private space and taking advantage of the outpatient waiting time.

**Table 1:** Example of the interview script

Sample Questions for Child	
• Can you tell me about when you knew that you had a disease and had to treat it?	
• Do you want to know more disease information?	
• How do you want to know about the disease?	
• What do you want to know about the disease?	
Sample Questions for parents	
• Can you tell me when you told your child about the disease?	
• What do you think about telling your child about the disease?	
• (When telling your child about the disease) How do you think you should tell?	
• (When telling your child about the disease) What do you think you should tell?	

### Ethical considerations

The Research Ethics Boards at St. Luke’s International University (19-A011) provided ethics approval. Research commenced after approval. With participants’ permission, we audio-recorded throughout the interview using the recorder. Interviews were carefully conducted to ensure that participants were not harmed, and we created an environment that allowed them to decline even if the interview session was in progress.

### Data analysis

In this study, the research theoretical position was adopted was based on critical realism, which acknowledges how individuals make sense of their experiences while focusing on the material and other limits of reality [22]. We conducted the thematic analysis following the six steps developed by Braun and Clarke [19, 23]. First, the verbal data were transcribed into text data by the first author, using numbering for a word file to protect participants’ anonymity. The authors (N.Y. and Y.N.) and three master’s students read all word lists to be familiar with the data and recorded noticeable aspects. We did this by hand initially, working through hard copies of the transcripts. Second, we coded the data using a semantic approach into meaningful contexts. An example of the coding process is displayed in Table 2. Then, we uploaded and analyzed approximately 80% of the data with the support of the qualitative analysis software NVivo. We excluded data not directly related to the research question, such as information needs at the time of the interview. Third, we looked for patterns and grouped similar codes as preliminary themes. Fourth, preliminary themes and codes were carefully reviewed and modified to ensure consistency with the research questions. Fifth, we defined themes to summarize the meaning and named them. We repeated these processes and revised identified themes to finalize themes to find information needs for children with leukemia. A third party, K.K., advised and refined the themes to improve the analysis’s validity. Disagreements were resolved through discussion. A native English speaking nursing professor made further revisions to clarify the English expressions of the categories and sub-categories. Sixth, we wrote up these findings.

**Table 2:** Example of the analysis process

Interview data extract	Codes	Sub-themes	Themes
*“I think it is a joke when my mom says it,* ***it is more believable to hear the disease from the doctor****.”* (11-year-old child)	**Need to communicate with the most appropriate person**: Perceptions of children and parents that they need to communicate with the most appropriate person	**How to tell**	**Beginning to cope**
*“****If the doctors tell us about the disease, we can ask them****.* ***Even if I did not understand something, they would answer us.*** *If parents tell something to their children, we are affected by our own thoughts. However, the doctor will tell children about the diseases directly. If we do not know, they can answer it. I was convinced by looking at my younger child’s attitude.”* (Parent of a 13-year-old child)			
*“****The cartoon is interesting.*** *For example, it is easy to understand abrasions. If you get hurt, the white blood cells kill the bacteria, the platelets come, and the fibrin seals it up.* ***I think it is very understandable****. (At diagnosis) It was not easy to understand the disease. But if there was a “Cells at Work (cartoon)” at that time, I think I could understand.”* (9-year-old child)	**Use communication tools with pictures and stories**: perceptions of children and parents who need tools to communicate leukemia-related information		
*“I have the picture books at home. There are some picture books, and one of the books is drawn about bone illness. So* ***I think it’s better to make a picture book about leukemia****.”* (8-year-old child)			
*“****It is essential to say that this (cancer) attacks or changes using the pictures****. We can see it in the picture. If you are an adult, you will draw a picture in your head when you think about leukemia. But children cannot do that.* ***Even my younger child could understand the name of the disease and my older child’s condition****.”* (Parent of a 13-year-old child)			

## Results

### Characteristics of participants

We recruited 13 parents; four parents decided not to participate (three parents were not interested in the study, and one did not have enough time). One refused the child’s participation because she did not want her child to talk about the illness. For an unknown reason, one child refused to participate. In total, seven children and nine parents (mothers = 8, father = 1) participated in this study (Table 3). All children (3 boys; 4 girls, median age at interview was 9 years, range: 7-13 years, mean duration from diagnosis was 2.9 years) were diagnosed with acute lymphoblastic leukemia at the median age of 4 (range: 3-10 years). Three were undergoing treatment, one had relapsed at the time of the interview. All had received leukemia information, including the name of the disease.

**Table 3:** Characteristics of participants

**Children with leukemia (*N*** **= 7**)	
	Median (range)
Age at interview	9 (7-13)
Age at diagnosis	4 (3-10)
Sex	n (%)
Boy	3 (42.9)
Girl	4 (57.1)
Treatment status	
Undergoing	3 (42.9)
Completed	4 (57.1)
Relapse	
No	6 (85.7)
Yes	1 (14.3)
Education	
Elementary school	6 (85.7)
Junior high school	1 (14.3)
**Parents (*N*** **= 9)**	
	Median (range)
Age at interview	41 (37-47)
Role of parents	n (%)
Mother	8 (88.9)
Father	1 (11.1)
Employment status	
Employed	4 (44.4)
Unemployed	4 (44.4)
No answer	1 (11.1)

### Themes

We identified three themes consisting of seven sub-themes for the information needs of children: 1) beginning to cope, 2) avoiding disclosure – protecting child, and 3) informational support (Table 4).

**Table 4:** Information needs of children with leukemia

Themes	Sub-themes	Codes
**Beginning to cope**	Why tell	Anxiety due to lack of information
		Unconvinced without information
		Need to understand the disease
		Need to understand the reason for hospitalization
	When to tell	Need to communicate at the beginning
		Need to communicate with emotional leeway
		Need to communicate with time leeway
	How to tell	Use suitable amount of information
		Need to communicate with the most appropriate person
		Use communication tools with pictures and stories
**Avoiding disclosure – protecting child**	Too complicated	Avoid incomprehensible information to children
	Too upsetting	Avoid the word “cancer”
		Avoid saying a life-threatening condition
**Informational support**	Making sense of it all	Information about the disease
		Information about treatment
		Information about hospitalization
		Information about infection prevention measures
		Information about side effects
	‘Upside’ of leukemia	Benefits of hospitalization
		Treatment outcomes
		Family support

### Theme 1: beginning to cope

The first theme referred to children and their parents’ perception that gaining leukemia information was necessary to cope with the disease. The children expressed emotions such as shock and surprise at the diagnosis; inevitably, all children needed to receive leukemia information. We identified three sub-themes, a) why tell, b) when to tell, and c) how to tell.

#### Why tell

The children and their parents reported that children needed information to understand why they have to cope with leukemia. The children stated that if they did not receive leukemia information, they would feel “sad” and expressed they would feel sorry for themselves. One child expressed:

*“[If no one tells me] I would feel sorry for myself. I do not know what is wrong with me, so I do not understand why I was sick.” (7-year-old child)*.

The children indicated that if they were not given any information, they would ask for it to relieve their anxiety. Another child stated:

*“Even if someone does not tell me, I would be concerned and ask [for] it.” (11-year-old child)*.

The parents also believed that cancer treatment was painful and long-term, and their child must understand they had the disease. One parent declared:

*“I thought, illnesses are hard to treat if the child does not want to get well.” (Parent of a 13-year-old child)*.

Most of the parents had to explain the disease to their children because they could not force them to undergo treatment or be hospitalized and they needed to engage the child’s cooperation. As one parent expressed:

“*I had no choice. It is necessary to explain why because I know he does not want to do it [go into the hospital].*” *(Parent of an 8-year-old child).*

Many parents were told by the physician that open communication with their child about leukemia was necessary; so, they decided to tell their children about their leukemia. Another parent stated:

*“The doctor told me that I should tell my child about the illness because he/she is old enough to understand, so I did.” (Parent of an 11-year-old child)*.

#### When to tell

The children reported they needed to know about leukemia at the time of diagnosis. One child revealed:

*“I want to know before I go into the hospital to understand why I am in the hospital. It is because I might be surprised if I am going to be in the hospital all of a sudden.” (7-year-old child)*.

In contrast, some parents thought that the time of diagnosis was not a suitable time to tell their children about leukemia. Some parents had difficulty accepting that their child had leukemia and did not feel like telling them. Suppose they could not grasp why their child was diagnosed with leukemia. In that case, it was challenging to prepare to share that information. One parent shared:

*“I had to accept the disease, and I did not immediately think of how to explain it to my child, who had just started kindergarten yet.” (Parent of a 9-year-old child)*.

Some parents explained that the process of their child’s admission to the hospital was so fast-paced that they did not have enough time to explain what was happening to the child. Another parent revealed:

*“*I *did not understand what was going to happen? We heard that our child might have cancer on Wednesday, and then, the next Tuesday, was admitted to the hospital.” (Parent of an 8-year-old child)*.

When some children were admitted to the hospital, they were in poor condition due to the symptoms of leukemia. There were repeated examinations, such as blood tests and bone marrow punctures and a parent noted that:

*“At the time of the hospitalization, our child’s condition was fuzzy and sluggish because a bone marrow sample had been taken. Moreover, our child was lonely because going home was not an option.” (Parent of an 8-year-old child)*.

Moreover, some parents reported that they had to understand leukemia before telling children. As one parent mused:

*“I wonder if telling children about leukemia would be difficult if parents did not understand it.” (Parent of a 13-year-old child)*.

#### How to tell

The children and their parents talked about the need of how information was communicated to them The children reported that if the information was given little by little, they cannot understand it very well and desired to know a summary of leukemia information as the overall big picture. For example, one child expressed:

*“It is better to explain about leukemia all at once, not in little bits and pieces. It is easier to understand if you do it all together.” (8-year-old child)*.

While parents thought that children could not sort through a huge amount of information at once. One parent exclaimed:

*“(It is better to explain each time) Even if we explain it altogether at once, the child cannot understand.” (Parents of an 8-year-old child)*.

The children and their parents respected physicians as HCPs who had knowledge about diseases and believed that they were the best ones to explain. Another child thought:

*“It is more believable to hear about leukemia from the doctor.” (11-year-old child).*

One parent also affirmed:

*“If the doctors tell us about leukemia, we can ask them. Even if I didn’t understand something, they would answer us.” (Parent of a 13-year-old child)*.

Yet, one of the parents thought that the mother was the best person to explain leukemia because she communicates well with her child. She declared,

“*I think I am the best person to tell my child at first. I am more appropriate than father.*” *(Parent of an 11-year-old child)*.

The parents realized that their child understood leukemia by using communication tools such as picture storyboards, pictures, illustrated books, and cartoons. One parent contemplated.

*“Suppose it is an easy-to-understand cartoon that I think is just right. If I read it saying it is interesting then I wonder if I talked about cancer after studying it for a while it will help my child understand.” (Parent of an 11-year-old child)*.

The children suggested that they could still understand leukemia even if they were younger than 7 years old if they used easily accessible and interesting strategies such as pictures and cartoons. Some children read picture stories, picture books, and cartoons to help them understand how the body works and what the disease is. As one child pointed out:

“*It would be easier to understand if there are pictures or something like that.” (13-year-old child)*.

### Theme 2: avoiding disclosure – protecting the child

The second theme referred to the perception that parents should protect their child by avoiding the disclosure of information to their child that would be upsetting. We identified two sub-themes related to information disclosure: a) too complicated, and b) too upsetting. Although the children did not mention that any information should be avoided, parents thought that they should avoid the information that they determined would be challenging for their children to understand and accept.

#### Too complicated

Some parents believed that if their child was diagnosed with leukemia as a preschooler, they could not understand it. The parents thought their child’s understanding was too limited. They believed that their child could recognize only the symptoms but not the disease. One parent stated:

*“When the child was a toddler, he/she could not understand the disease, so I had to tell the child what his/her existing symptoms were rather than the name of the disease.” (Parent of a 9-year-old child)*.

#### Too upsetting

The parents reported that it was excruciating for them to tell their children something they thought would cause unnecessary distress. These contents included the word cancer and life-threatening condition. One parent declared:

“*I told the doctors that I did not want them to say cancer. I told them that I would tell them when I was able to say it during treatment.” (Parent of an 11-year-old child)*.

Another parent affirmed:

*“I think it is better not to say anything life-threatening.” (Parent of an 8-year-old child)*.

### Theme 3: informational support

The third theme referred to the types of information they needed to know for coping with leukemia. We identified two sub-themes, a) making sense of it all and b) ‘upside’ of leukemia.

#### Making sense of it all

The children reported that they needed to know information about making sense of their situation, such as the name of their disease, including the term leukemia, treatment and hospitalization. One child articulated:

*“If I did not know the name of the disease, I would not understand what it was later.” (7-year-old child)*.

The children needed to know about the disease condition, but the degree they needed to know differed from individual to individual. Some children desired to know the role of cells to understand the disease process. A child explained:

*“I think it would be good to tell children about the active cells and bacteria in their bodies first and then talk about the disease.” (9-year-old child)*.

Some children just focused on recovering from leukemia and did not need further information because they received all the information they wanted. As a young teenage noted:

*“I just wanted to recover from leukemia, and I received everything I needed to know. So, I did not need more information.” (13-year-old child)*.

The parents focused on having a disease rather than the name of the disease and clinical condition. As an example, one parent mentioned:

“*I did not say leukemia, and I said it is a blood disease.*” *(Parent of a 9-year-old child)*.

If the child had never encountered a severe illness before, they only knew that they had leukemia, but could not understand why they had to be hospitalized. Children needed to know that treatments and hospitalization were necessary to recover from leukemia. This was reflected by this child’s statement:

*“The only thing I could think of was, that I understood I had a sickness, but why did I have to be there anyway? The reason should have been made clear.” (9-year-old child)*.

Some parents reported that they had no choice but to tell their child what they had to do and what was required. They needed to talk about long-term hospitalization, infection prevention measures and side effects. A reality of the parent’s situation was highlight by their explanation:

*“My child had to be hospitalized, so I told my child right away. I said it would be a long stay. I had to go back home because I had a baby.” (Parent of a 13-year-old child)*.

#### ‘Upside’ of leukemia

The children needed to know the benefits of having leukemia as well as the bad news. For them, the benefits were making friends, learning more about the disease, and staying out of the hospital. One young child exclaimed,

“*It would help if you also told children good things. For example, they can learn about the disease and make friends.*” *(7-year-old child)*.

The parents wished to communicate information that would give their child a sense of security first, in that the targets and outlook for the treatments looked promising, and then emphasized that their hearts are always together, despite the physical distance. One parent reported,

*“I wish I could have told my child about the [treatment] prospects during hospitalization.*” *(Parent of an 8-year-old child).*

Another parent uttered that,

“*We need to tell children that [the hearts of] our family, father, mother, and siblings are all together.*” *(Parent of a 10-year-old child)*.

## Discussion

In this study, we examined the information needs of children with leukemia from both the children’s and their parents’ perspectives at diagnosis. We also revealed similarities and differences between children and their parents. Communication is one of the essential elements of Child and family-centered care (CFCC). The findings increase our knowledge and would help HCPs communicate with children with leukemia and their families.

In Asian cultures has been known indirect truth-telling is more culturally accepted [24]. However, the idea that even children need to be told the truth has permeated the culture, and in the past 60 years, the style of cancer communication has become more forthcoming [25]. Previous studies in Western countries have shown that children want to be informed honestly and openly [10, 21] from the outset of diagnosis [21, 26]. In this study, the children believed that cancer-related information was needed to cope with cancer. Although children’s information needs vary, they needed clear information, including the disease, treatment, hospitalization, and advantage of hospitalization from the beginning of diagnosis. As previous studies have shown, the children need to understand the disease, the treatment, and hospitalization [20, 27], while maintaining hope [21]. When communicating with children, HCPs must be aware of the following: children who had never had a severe illness or were hospitalized complained that they could not understand the illness and the need for treatments. It is not enough to explain that children have diseases and have to take treatment. When telling a child about cancer, HCPs need to explain what is happening in children’s bodies and why treatments are needed so that they can understand it. They needed cancer-related communication using comprehensive and engaging tools with physicians, and HCPs must respect children’s preferences.

The parents had different perceptions about children’s information needs. Previous studies showed that parents struggle to control their feelings at diagnosis [7, 28]. If they are shocked by the diagnosis, they may not communicate enough [28] or decide not to tell their child [29]. In this study, although parents believed that cancer-related information is necessary for children to understand the situation, they thought that they should manage communication with children about the disease to avoid their distress. Some parents were shocked by their child’s cancer diagnosis, and they felt they should not tell their children about the disease. Previous work has pointed out that communication with children is greatly influenced by parents’ perceptions and beliefs about cancer when talking to children about life-threatening conditions [7, 30]. HCPs need to understand parents’ perceptions to communicate with their children about leukemia effectively. Parents felt that children should not be informed about what they considered to be too complex for children to understand and to distressful (e.g., the word “cancer,” life-threatening, and invasive procedures). As previous studies have shown [31, 32], the parents believed that managing the information was necessary to protect their children from the psychological burden of distress. However, parent-centered communication might lead to disempowering children. Children desire to be respected as patients and to have their information needs addressed [4]. Thus, HCPs should explore the motivations behind parents’ attitudes against communication with children and provide them with sufficient support so as to improve CFCC. HCPs should tell parents that effective communication helps children cope with leukemia, and involve children in the healthcare discussion.

### Study strength and limitations

A strength of this study is its focus on the perspective of both children with leukemia and their parents. We conducted the interviews individually and analyzed the data using a semantic approach. This approach allowed us to understand the children and their parents’ views deeply, and the findings would be used to improve CFCC.

However, there are some limitations to this study. First, we decided to include only children over 7 years old and could not involve pre-school children. Second, our study had a small sample size and could not find differences by gender or age. Third, we included children who passed the time from diagnosis and were at different treatment points. Recall bias might have occurred, and the information needs of the child and their parents may differ from those at the time of diagnosis. Fourth, all participants had already been told about leukemia. We could not involve children who had not received any explanation about leukemia.

### Implications for future research and clinical implications

Children and their parents might have different opinions, and HCPs should communicate with each other to respect children’s information needs. Although the research should be conducted while protecting children’s human rights. It is essential to design research to understand children’s views. In this study, we could not involve pre-school children, children who had not received an explanation about cancer, and children and their parents immediately after diagnosis. We recommend that future research include these participants to understand their views. In addition, further research is needed to investigate the differences in information needs by patient demographics, such as age and gender.

## Conclusion

This study derived information needs of children with leukemia at diagnosis from children and parents’ perspectives. Children need explanations that can make sense to them. However, parents might manage communication with children about the disease to avoid their own distress as well as the child’s distress. HCPs need to understand the parents’ suffering and communicate with them involving their children to respect the child’s preferences.

## Data Availability

The datasets generated and analyzed during the current study are not publicly available due to individual privacy and the sensitive nature of the interviews but are available from the corresponding author on reasonable request.

## Abbreviations

CFCC: Child- and Family-Centered Care
HCPs: Healthcare Professionals
